# Large-Scale Protein-Protein Interaction Analysis in Arabidopsis Mesophyll Protoplasts by Split Firefly Luciferase Complementation

**DOI:** 10.1371/journal.pone.0027364

**Published:** 2011-11-09

**Authors:** Jian-Feng Li, Jenifer Bush, Yan Xiong, Lei Li, Matthew McCormack

**Affiliations:** 1 Department of Genetics, Harvard Medical School, Boston, Massachusetts, United States of America; 2 Department of Molecular Biology, Massachusetts General Hospital, Boston, Massachusetts, United States of America; Ohio State University, United States of America

## Abstract

Protein-protein interactions (PPIs) constitute the regulatory network that coordinates diverse cellular functions. There are growing needs in plant research for creating protein interaction maps behind complex cellular processes and at a systems biology level. However, only a few approaches have been successfully used for large-scale surveys of PPIs in plants, each having advantages and disadvantages. Here we present split firefly luciferase complementation (SFLC) as a highly sensitive and noninvasive technique for *in planta* PPI investigation. In this assay, the separate halves of a firefly luciferase can come into close proximity and transiently restore its catalytic activity only when their fusion partners, namely the two proteins of interest, interact with each other. This assay was conferred with quantitativeness and high throughput potential when the Arabidopsis mesophyll protoplast system and a microplate luminometer were employed for protein expression and luciferase measurement, respectively. Using the SFLC assay, we could monitor the dynamics of rapamycin-induced and ascomycin-disrupted interaction between Arabidopsis FRB and human FKBP proteins in a near real-time manner. As a proof of concept for large-scale PPI survey, we further applied the SFLC assay to testing 132 binary PPIs among 8 auxin response factors (ARFs) and 12 Aux/IAA proteins from Arabidopsis. Our results demonstrated that the SFLC assay is ideal for *in vivo* quantitative PPI analysis in plant cells and is particularly powerful for large-scale binary PPI screens.

## Introduction

The function of a protein in living plant cells is typically carried out and tightly modulated through interactions with other proteins including cognite substrates, scaffolding proteins and activity or stability regulators. Meanwhile, a large number of proteins need to dimerize or form higher-order oligomers for proper function [1]. It is the integrative network of all protein-protein interactions (PPIs) in the cell that virtually determines its developmental fate as well as its responses to the ever-changing extracellular environment. Understanding of the PPI network is thus crucial for elucidation of the molecular mechanisms underlying complex cellular processes such as signal transduction. To date, many approaches for PPI analysis have been applied in plant research [2], [3]. However, only a few of them, including yeast two-hybrid (Y2H), affinity purification combined with mass spectrometry (AP-MS), bimolecular fluorescence complementation (BiFC) and protein microarray, have been successfully used for large-scale PPI studies [4]-[8]. Despite the potential of high throughput, these techniques each have a few drawbacks [2], [3]. Y2H and its improved derivatives, as heterologous systems, may lack plant co-factors or subcellular compartments necessary for specific PPIs, leading to false positive and negative results. Protein microarray is conducted *in vitro* under non-physiological conditions without any spatial and temporal control of protein expression, and thus is inevitably associated with a high false positive rate. AP-MS aims at identifying protein complexes and is inherently unable to distinguish between direct and indirect PPIs. Also, cell lysis prior to affinity purification can disrupt weak PPIs while creating artificial ones between those proteins which have no chance to co-localize in intact plant cells. Although BiFC allows the visualization of subcellular localizations of PPIs, it can not reflect the dynamics of a given PPI in a real-time manner due to the irreversible reconstitution and slow maturation of the fluorescent protein. Since the external light source used in the BiFC assay will also excite the autofluorescence of plant cells, BiFC signal detection needs to be carried out with considerable caution, making the assay not ideal for high-throughput performance. Therefore, a novel approach that could study *in vivo* PPIs in plant cells with the promise of high-throughput application would be highly valuable to the plant research community.

The split luciferase complementation assay was originally explored in mammalian research as a new tool for PPI investigation [9], [10]. In this assay, two proteins of interest are respectively fused to the two halves of a luciferase (e.g., *Renilla* luciferase or firefly luciferase). If the interaction between these two proteins occurs, it would bring the two luciferase fragments into close proximity and transiently restore the catalytic activity of luciferase. Since the substrate of luciferase can penetrate the cell membrane, this assay allows a noninvasive investigation of PPIs in mammalian cells and whole animals. Recently, the split *Renilla* luciferase complementation assay has been adapted into plant research [11]. However, the widely used *Renilla* luciferase substrate coelenterazine is very labile due to its spontaneous oxidation [11]. The use of coelenterazine may also pose an unpredictable impact on plant cell physiology since this chemical is actively transported by the P-glycoproteins on the plasma membrane [12], which are involved in phytohormone auxin transport [13]. Unlike coelenterazine, the firefly luciferase substrate D-luciferin remains stable in culture medium over days [14] and its cell penetration does not rely on the P-glycoproteins [12]. Moreover, firefly luciferase has the highest quantum yield during catalysis among all known bioluminescent enzymes [15], which could facilitate the signal detection at both cellular and tissue levels. A recent study has found no cytotoxicity induced by either firefly luciferase overexpression or D-luciferin treatment [16]. These features make the split firefly luciferase complementation more ideal for detecting *in vivo* PPIs in plant cells than the split *Renilla* luciferase complementation.

A firefly luciferase complementation imaging (LCI) assay and, very recently, a floated-leaf firefly luciferase complementation imaging (FLuCI) assay have been reported in plant research [17], [18]. Here we present a split firefly luciferase complementation (SFLC) assay which has two major differences from the LCI or FLuCI assay. First, our assay adopted different firefly luciferase fragments in complementation, the combination of which has been shown to generate higher signal-to-background ratio in two independent head-to-head comparisons with that used in the LCI or FLuCI assay [19], [20]. Second, instead of using a charge-coupled device (CCD) imaging system, our assay took advantage of the microplate luminometer for luciferase measurement. These differences dramatically increased the sensitivity and high throughput potential of our assay, making it more suitable for large-scale PPI analysis. As a proof of concept, we conducted a systematic binary PPI survey among 8 auxin response factors (ARFs) and 12 Aux/IAA proteins from Arabidopsis, and our data further supported the current auxin signaling model in terms of the response specificity regulation.

## Results

**Figure 1:**
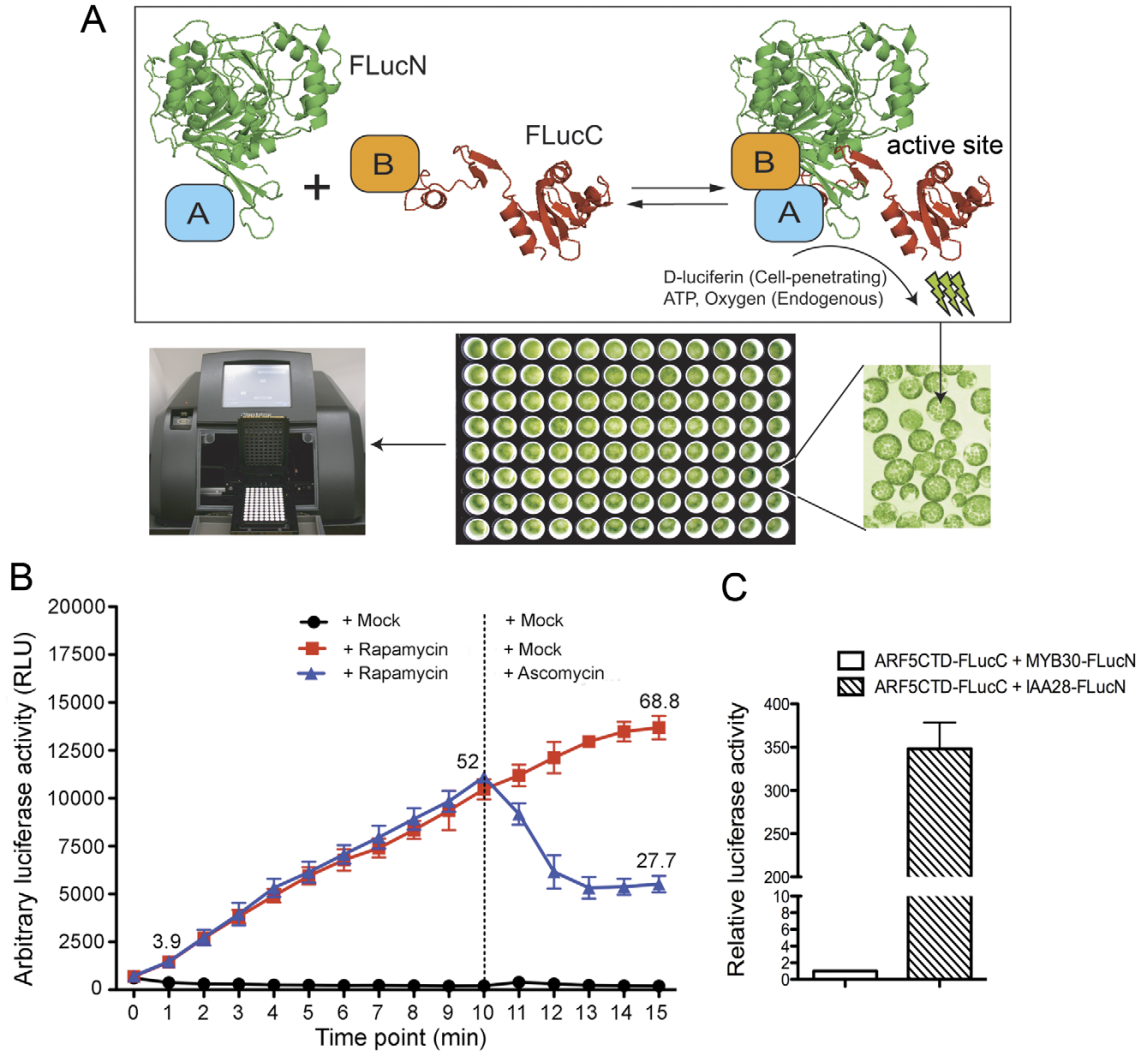
The split firefly luciferase complementation (SFLC) allows quantitative detection of *in vivo* protein-protein interaction in Arabidopsis mesophyll protoplasts. (A) Schematic diagram of the SFLC assay. Two fusion proteins, A-FLucN and B-FLucC, are co-expressed in Arabidopsis protoplasts. FLucN and FLucC indicate the N-terminal fragment (aa1-398, colored in green) and the C-terminal fragment (aa394–550, colored in red) of firefly luciferase, respectively, which are used in complementation. A and B stand for the two proteins for interaction test. An interaction between A and B would bring the two halves of firefly luciferase into close proximity and transiently restore its activity. This in turn leads to light emission upon substrate (D-luciferin) oxidation, which can be detected using a microplate luminometer in a noninvasive, quantitative and high-throughput manner. The location of the restored enzyme active site during SFLC is indicated. Note that the reconstitution of firefly luciferase in the SFLC assay is reversible. (B) Rapamycin-induced and ascomycin-disrupted interaction between Arabidopsis FRB (AtFRB) and human FKBP (HsFKBP) proteins monitored by the SFLC assay. Arabidopsis protoplasts co-expressing FLucN-AtFRB and FLucC-HsFKBP for 6 hr were divided into three equal aliquots (black, blue, and red), two (blue and red) treated with 10 nM rapamycin and one (black) treated with the solvent DMSO. At the time point of 10 min, one rapamycin-treated aliquot (blue) was treated with 10 µM ascomycin while the other two (red and black) were treated with DMSO. The restored luciferase activity at the indicated time points was recorded by a luminometer and the plateaued restored luciferase activity after 15 min rapamycin (or 5 min ascomycin) treatment was not shown. (C) Interaction between the auxin response factor 5 (ARF5) and IAA28 detected by the SFLC assay. The C-terminal domain (CTD) of ARF5-FLucC (ARF5CTD-FLucC) was co-expressed with IAA28-FLucN or MYB30-FLucN in protoplasts for 6 hr before recording the restored luciferase activity. Three biological replicates were conducted for (B) and (C).

Firefly luciferase (FLuc) fragments, aa 1-398 (FLucN) and aa 394-550 (FLucC)(Figure 1A), were used for complementation in our SFLC system because they generated negligible background and consequently ameliorated signal-to-noise ratio during complementation [19], [20]. Therefore, we cloned the coding sequence of FLucN into the transient expression vector pAN containing a double *35S* promoter and a *Nos* terminator to obtain the pcFLucN or pnFLucN plasmid (Figure S1). Similarly, the coding sequence of FLucC was cloned into the same vector to generate the pcFLucC or pnFLucC plasmid (Figure S1). The resulting plasmids contain multiple single-cut restriction enzyme sites to facilitate a subsequent insertion of any gene of interest in frame with the FLucN or FLucC coding sequence at either end. The compatible cohesive ends between *Spe*I/*Nhe*I/*Xba*I or *Bam*HI/*Bgl*II offer additional options in cloning the gene of interest in case some of these restriction sites exist in the gene. The coding sequence for a flexible linker, namely a double GGSGG peptide, was introduced between the inserted gene and the coding sequence of luciferase fragment (Figure S1) to minimize the interference between the two polypeptide domains in the hybrid protein.

To test whether these FLucN and FLucC constructs could be used in SFLC for PPI analysis in plant cells, we applied them to monitoring the interaction between Arabidopsis FRB (AtFRB) and human FKBP (HsFKBP) proteins. Rapamycin-induced interaction between these two proteins has been demonstrated in Y2H and *in vitro* pull-down assays [21] but never *in planta*. We co-expressed FLucN-AtFRB and FLucC-HsFKBP proteins in Arabidopsis mesophyll protoplasts for 6 hr before the addition of 10 nM rapamycin. We found that the interaction between AtFRB and HsFKBP could be detected as early as within 1 min after rapamycin treatment, as reflected by a 3.9-fold increase of the restored luciferase activity compared to the mock (i.e., DMSO) treatment (Figure 1B). Steady increase of the restored luciferase activity continued until the activity plateaued at 68.8-fold relative to the mock treatment after 15 min rapamycin treatment (Figure 1B). Ascomycin competes with rapamycin for HsFKBP binding and could thus interfere with the rapamycin-mediated interaction between FRB and FKBP [22]. Indeed, when 10 µM ascomycin was added after 10 min rapamycin treatment, an instant reduction of the restored luciferase activity occurred within 1 min and the activity quickly declined within 3 min from 52-fold to a minimum of 27-fold relative to the mock treatment (Figure 1B), where rapamycin and ascomycin probably reached equilibrium in HsFKBP binding. These data suggested that our SFLC assay can be used to monitor a dynamic PPI in plant cells in a near real-time manner with good inducibility and reversibility.

We next utilized the SFLC constructs to reproduce the positive interaction between Arabidopsis transcription repressor IAA28 [23] and transcription activator ARF5 [24] which has been demonstrated in Y2H assay [25]. We only used the C-terminal dimerization domain (CTD) of ARF proteins in the PPI tests in this work because: (i) the CTD of ARFs is sufficient for interaction with Aux/IAA proteins and with other ARF proteins [26]; (ii) since the CTD of ARFs was predominantly used for PPI tests in Y2H [26]-[28], for comparison purposes, it is desirable to use the same domain of ARFs in SFLC; (iii) despite the loss of nuclear localization sequence (NLS) in the ARF5 CTD, a significant fraction of this protein could still be detected in the nucleus when expressed as a mCherry fusion (ARF5CTD-mCherry, about 38 kDa) in Arabidopsis protoplasts (Figure S2) presumably due to its small size. By inference, ARFCTD-FLucC fusion (less than 30 kDa) should be prone to diffusing into the nucleus. In the SFLC assay, the fusion orientation of the luciferase fragment was determined based on the consideration that the putative interface between ARF and Aux/IAA is located at their C-terminus [26]. When we co-expressed IAA28-FLucN and ARF5CTD-FLucC proteins in Arabidopsis protoplasts for 6 hr, a strong restored luciferase activity could be readily detected by a luminometer (Figure 1C). This luciferase activity was approximately 350-fold higher than that of the negative control where IAA28 was replaced by MYB30, a transcription factor that is not expected to interact with ARF5 (Figure 1C). Again, these results suggested that our SFLC system is capable of quantitatively detecting *in vivo* PPI in plant cells.

**Figure 2:**
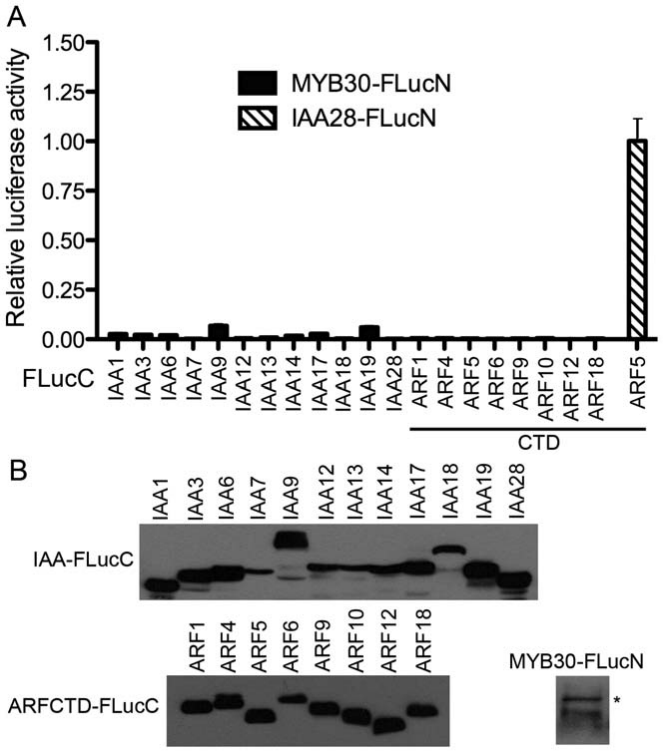
The split firefly luciferase complementation has a low background in Arabidopsis mesophyll protoplasts. (A) Only marginal restored luciferase activity could be detected in protoplasts co-expressing individual Aux/IAA-FLucC or ARF C-terminal domain-FLucC (ARFCTD-FLucC) with the unrelated protein MYB30-FLucN. Three biological replicates were conducted for each protein combination and similar interaction patterns were obtained. (B) The 12 Aux/IAAs, the C-terminal domain (CTD) of 8 ARFs, and MYB30 were all expressed in protoplasts when fused with FLucC or FLucN as indicated. Equal amounts of protoplasts (4×10^4^ cells) expressing 20 µg of individual construct for 6 hr were lysed in SDS-PAGE loading buffer. FLucC and FLucN fusion proteins were visualized by western blot using anti-FLucC and anti-FLucN antibodies, respectively. MYB30-FLucN in western blot is marked by an asterisk.

In principle, the use of protoplast expression system and microplate luminometer would confer high throughput potential to our SFLC assay. We next attempted to explore the feasibility of using our SFLC system in large-scale PPI analysis and to streamline the working protocol. We chose the interactions between Arabidopsis ARF and Aux/IAA families for testing because: (i) it is of great significance to understand these interactions in that they determine the specificity of auxin signaling [26], and (ii) current knowledge about these interactions was mostly obtained from heterologous PPI assays such as Y2H [26]–[28]. We selected 12 Aux/IAA and 8 ARF proteins to make 96 binary interaction tests using a 96-well microplate. The selected 12 Aux/IAAs include IAA1, IAA3, IAA6, IAA7, IAA9, IAA12-14, IAA17-19 and IAA28, whereby we purposely chose some phylogenetically related members (e.g., IAA12 and IAA13, [29]) to compare their interaction patterns with the ARFs. The 8 ARFs are ARF1, ARF4-6, ARF9, ARF10, ARF12 and ARF18, which were chosen from different branches on the phylogenetic tree of the ARF family [29] and likely bear distinct properties in interacting with the Aux/IAAs. Prior to performing the large-scale PPI tests, we first assessed the suitability of our SFLC assay for high-throughput screening (HTS) by calculating its Z-factor. The Z-factor is reflective of both the signal dynamic range and the data variation associated with the signal measurements in a given HTS assay, and therefore is a quantifiable parameter for assay quality evaluation [30]. For this purpose, we expressed the entire set of negative controls, namely individual IAA and ARFCTD candidates fused with FLucC in combination with MYB30-FLucN in Arabidopsis protoplasts. Although all these proteins were expressed in protoplasts (Figure 2B), their co-expression only yielded ignorable or marginal luciferase activities when compared with the co-expression of the positive control combination ARF5CTD-FLucC and IAA28-FLucN (Figure 2A), suggesting that our SFLC system has a fairly low background. We calculated the Z-factor (see Materials and Methods) of our SFLC assay to be approximately 0.55, which is within the Z-factor range (>0.5, [30]) of a robust HTS assay, suggesting that our assay is suitable for large-scale PPI analysis. Thereafter, in order to compare different PPI intensities, the ARF5CTD-FLucC and IAA28-FLucN interaction was routinely carried out in parallel with other PPI tests, thus we could standardize the restored luciferase activity of any protein combination against that of ARF5CTD-FLucC and IAA28–FLucN to achieve a relative luciferase activity reflecting the relative PPI intensity.

**Figure 3:**
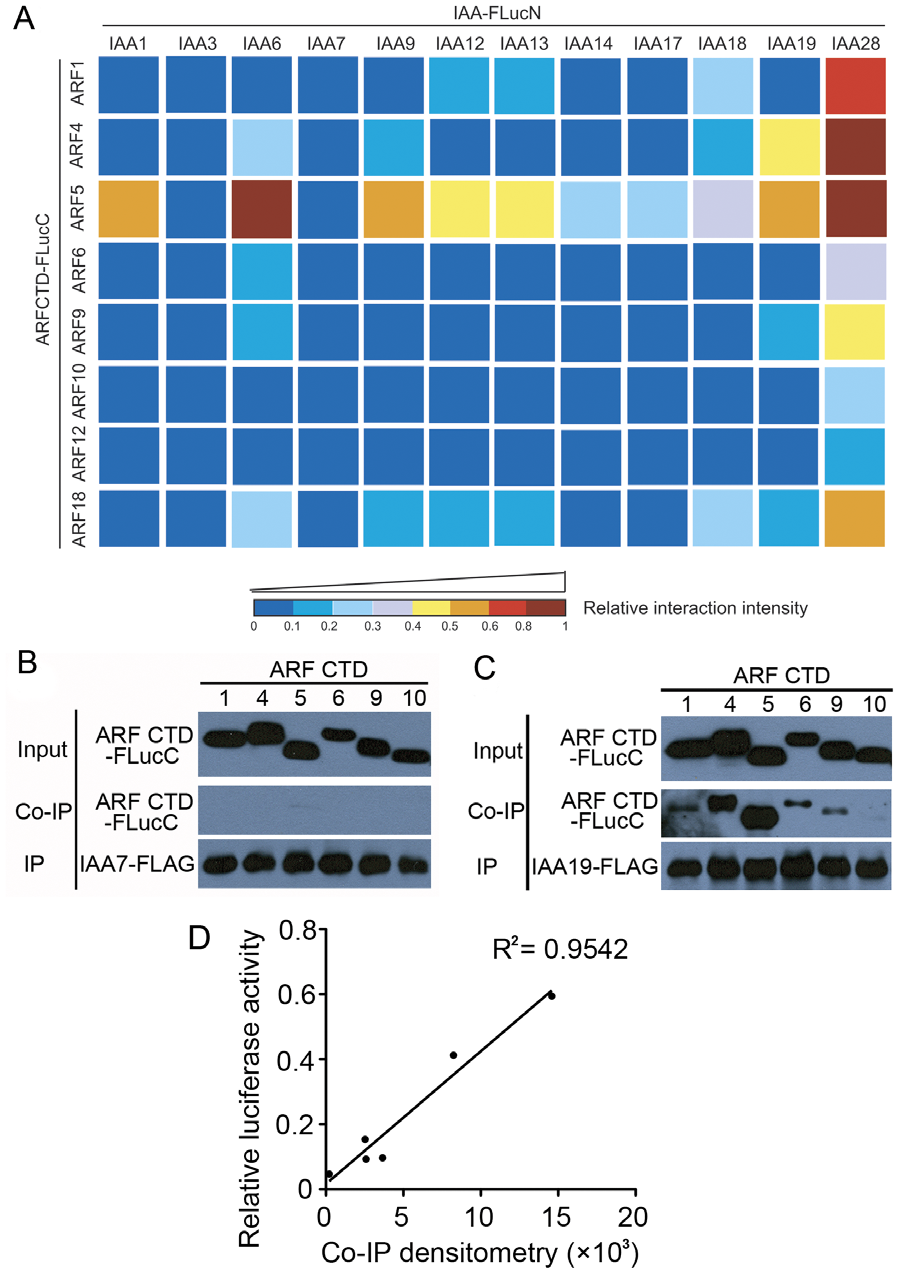
Binary interaction analysis between 12 Aux/IAAs and 8 ARFs by the SFLC assay. (A) The 96 different combinations between 12 Aux/IAA-FLucN and 8 ARFCTD-FLucC proteins resulted in huge differences in the restored luciferase activity, which reflected diverse PPI intensities. At least three biological replicates were assayed for each Aux/IAA-ARF combination and similar interaction patterns were obtained. Quantitative data are provided in Table S1. The relative interaction intensity of a given combination was generated by standardizing its restored luciferase activity against that of ARF5CTD-FLucC and IAA28-FLucN, and is presented in a heat map, with the coldest color (dark blue) indicating the lowest interaction intensity and the hottest color (dark red) indicating the highest interaction intensity. The color bar indicates the range of relative interaction intensity corresponding to each color. (B) None of the 6 well-expressed ARF CTD proteins could be co-precipitated with the IAA7-FLAG protein during co-immunoprecipitation. (C) Diverse amounts of the 6 ARF CTD proteins were co-precipitated with the IAA19-FLAG protein during co-immunoprecipitation. In (B) and (C), 100 µg of IAA7-FLAG or 20 µg of IAA19-FLAG construct was co-expressed with 100 µg of indicated ARFCTD-FLucC construct in an excessive amount of protoplasts (5×10^5^ cells) for 6 hr, and a modest amount (10 µl of 50% slurry) of anti-FLAG M2 agarose beads were used for immunoprecipitation in order to pull down comparable amounts of Aux/IAA proteins. (D) Positive correlation between the immunoblot signal from (C) determined by densitometric analysis using Image J program and the corresponding SFLC results from (A) and Table S1.

Next we co-expressed 12 Aux/IAA-FLucN and 8 ARFCTD-FLucC proteins in a pairwise manner in protoplasts. The resulting 96 different protein combinations have led to huge differences in the restored luciferase activities (Figure 3A and Table S1), suggesting their distinct PPI intensities. At least 3 biological replicates were performed for each Aux/IAA-ARF combination and similar protein interaction profile was obtained. Interestingly, the tested homologs in the Aux/IAA family [29], such as IAA12 and IAA13, IAA14 and IAA17, IAA6 and IAA19, demonstrated similar interaction patterns with the 8 ARF proteins. An unexpected exception was IAA7, which is phylogenetically related to IAA14 and IAA17 [29] but had barely detectable interaction with any of the tested ARFs. Likewise, IAA3 also exhibited subtle interactions with the selected ARFs. In contrast, IAA28 showed strong interactions with all the tested ARFs. On the other hand, the transcription activator ARF5 stood out among the tested ARFs to interact with 10 Aux/IAA proteins (Figure 3A), where 8 of these interactions have been found earlier by Y2H (Table S3)[25], [31]–[34] or fluorescence cross-correlation assay [35]. Unexpectedly, compared to ARF5, another transcription activator ARF6 [24] exhibited weaker interactions with those Aux/IAA proteins. The ARFCTD-FLucC proteins were expressed at a similar level in protoplasts (Figure 2B). However, we were unable to determine the relative levels of IAA-FLucN proteins by immunoblotting using the anti-FLucN antibody in the SFLC assay (data not shown). This difficulty was presumably caused by the poor affinity of the anti-FLucN antibody and the low levels of IAA-FLucN proteins as both the Aux/IAA proteins and the FLucN fragment could be intrinsically unstable. Indeed, we were able to detect the expression of all 12 IAA proteins when they were fused to FLucC (Figure 2B). Notably, IAA7 and IAA12 appeared to be less abundant while IAA3, IAA9 and IAA28 were accumulated at relatively higher levels when expressed in protoplasts. These protein abundances were in agreement with the previously reported *in planta* data [36]–[38], suggesting that the *35S* promoter-driven expression of individual *Aux/IAA* genes in protoplasts was still under proteolytic regulation as in whole plant.

To further confirm the SFLC data, we employed co-immunoprecipitation (co-IP) to double-check some of the interactions between Aux/IAAs and ARFs. The binary interactions between 2 Aux/IAAs (i.e., IAA7 and IAA19) and 6 ARFs (i.e., ARF1, ARF4-6, ARF9 and ARF10) were chosen to test since these two Aux/IAA proteins have shown dramatically different interaction patterns with individual ARF proteins in the SFLC assay. We co-expressed FLAG-tagged IAA7 or IAA19 with individual ARFCTD-FLucC in an excessive amount of protoplasts (5×10^5^ cells). Considering that the protein level of IAA19 is approximately 20 fold more than that of IAA7 (Figure 2B), a modest amount (10 µl of 50% slurry) of anti-FLAG M2 agarose beads were used for immunoprecipitation in order to pull down comparable amounts of Aux/IAA proteins (Figure 3B and 3C). Interestingly, none of the well-expressed ARF CTD proteins were co-precipitated with the IAA7-FLAG protein (Figure 3B). In contrast, large amounts of ARF4 and ARF5, moderate amounts of ARF1, ARF6 and ARF9 could be co-precipitated with IAA19-FLAG protein (Figure 3C). When we quantified the immunoblot signal for each ARF protein co-precipitated with IAA19 and compared with the corresponding SFLC results (Figure 3A and Table S1), a significant positive correlation (R^2^ = 0.9542) between these two parts of data was confirmed, affirming the reliability of our SFLC system.

**Figure 4:**
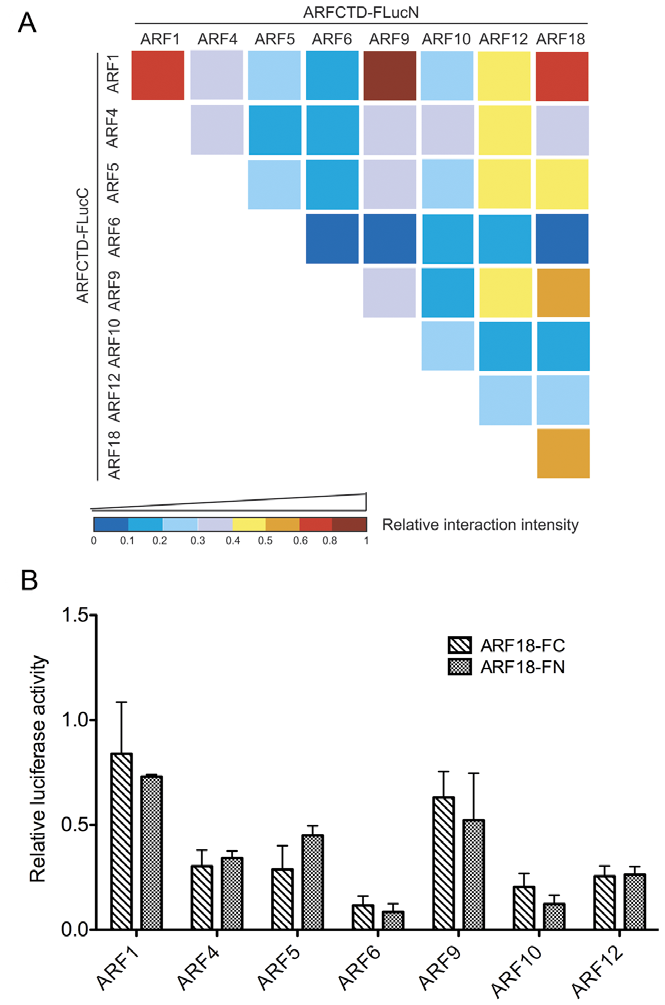
Homotypic and heterotypic interaction analysis between 8 ARFs by the SFLC assay. (A) Most (33/36) of the ARF-ARF combinations in the SFLC assay yielded detectable reconstituted luciferase activity, suggesting a ubiquitous occurrence of ARF dimerization. The relative interaction intensity of a given combination was generated by standardizing its restored luciferase activity against that of ARF5CTD-FLucC and IAA28-FLucN, and was presented in a heat map, with the coldest color (dark blue) indicating the lowest interaction intensity and the hottest color (dark red) indicating the highest interaction intensity. The color bar indicates the range of relative interaction intensity corresponding to each color. (B) The overall pattern of the PPIs between ARF18 and the other 7 ARFs remained similar to each other no matter whether ARF18 was chosen to fuse with FLucC or FLucN during complementation. At least three biological replicates were assayed for each ARF-ARF combination in (A) and (B) and similar interaction patterns were observed. Quantitative data are provided in Table S2.

The CTD of ARF proteins has been previously shown to dimerize not only with Aux/IAA proteins but also with other ARF proteins [26]. Next, we applied the SFLC assay to study the homo- and hetero-dimerization between the 8 ARF proteins. To this end, the ARFCTD-FLucC proteins were co-expressed with ARFCTD-FLucN proteins in a pairwise manner in protoplasts. Among the 36 different ARF combinations, 33 of them yielded detectable, albeit diverse, reconstituted luciferase activities (Figure 4A and Table S2), suggesting a ubiquitous occurrence of ARF dimerization in plant cells. At least 3 biological repeats were conducted for each ARF-ARF combination to make sure that the interaction pattern was reproducible. Stronger ARF dimerizations were found to involve ARF transcription repressors such as ARF1, ARF9 and ARF18. Interestingly, although ARF1 interacted strongly with other ARF repressors, it only weakly dimerized with the two ARF activators, ARF5 and ARF6. Moreover, ARF5 and, especially, ARF6 showed relatively weaker dimerization with themselves and with other ARF repressors when compared to ARF1. These results suggested that ARF-ARF interactions could also be highly selective. To characterize our SFLC system more thoroughly, we asked whether the switch of FLucN and FLucC between the two tested ARF proteins could influence the output of the SFLC assay. To address this question, we compared the restored luciferase activities of heterodimerizations between ARF18 and the other 7 ARFs where ARF18 was fused to FLucC and FLucN, respectively, during complementation. Although the restored luciferase activity of individual heterodimerization could slightly vary between the two strategies, the overall patterns of these 7 PPIs were consistent (Figure 4B). Our data suggested that fusion with either fragment of firefly luciferase only slimly affected the readouts of the SFLC assay in these cases.

## Discussion

### The SFLC assay is ideal for *in vivo* PPI studies in plant cells

In comparison with existing PPI assays, the SFLC assay provides several advantages: (i) This assay provides a noninvasive analysis of *in vivo* PPIs in plant cells since the substrate D-luciferin is cell permeable and other co-factors in the reaction such as ATP and O_2_ are supplied from endogenous pools. This merit is particularly important in that a PPI identified under undisturbed native conditions is more likely to be biologically relevant. (ii) This assay can quantitatively examine a large number of PPIs within 7–8 hr from protoplast transfection to data collection. The Arabidopsis protoplast system allows high-throughput DNA transfection, and the protoplasts prepared from eight to ten 4-week-old Arabidopsis plants are sufficient for over 140 PPI tests. In principle, the use of mesophyll protoplasts makes this assay transferable to other plant species (e.g., tobacco, maize, rice and switchgrass) or tissue types (e.g., root and seedling) supporting protoplast isolation [39], [40]. The use of luminometer enables a high-throughput and quantitative measurement of the restored luciferase activity. It should be mentioned that the SFLC assay described here can also be adapted for *in situ* PPI analysis in whole plant through bolistic bombardment, leaf infiltration or stable transformation. In those cases, the high permeability of the substrate D-luciferin into the deeper cell layers in roots, hypocotyl, and leaves including the shoot apical region [41] would facilitate PPI signal detection in intact tissues. (iii) The SFLC assay is able to monitor a dynamic PPI in a near real-time manner. During the assay, an active site of the firefly luciferase will be instantaneously re-formed once the interaction between the fusion partners of the two luciferase fragments brings them into proximity (Figure 1A). Conversely, loss of PPI between the fusion partners would immediately separate the two luciferase fragments and eliminate the transiently restored activity since the two fragments *per se* do not have direct contact to hold the complex (Figure 1A). This is different from BiFC where the reconstitution of a barrel-like fluorescent protein is strongly stabilized by more than 30 hydrogen bonds between its two halves [42]. (iv) The SFLC assay has great sensitivity in detecting PPI. The deliberately selected luciferase fragment combination, the absence of luciferase-analogous enzymes in plant cells and no need for an external light source in the assay all together help maintain an extremely low background in protoplasts. Thus, even a marginal amount of restored luciferase activity could be readily detected, making the SFLC assay well suited for examining weak interactions between low-abundance or unstable proteins such as Aux/IAAs.

While the SFLC assay offers a number of advantages, it also comes with a few general limitations that may reduce its usefulness in certain situations. For example, although the SFLC assay is capable of visualizing PPI at the tissue level through CCD imaging [17], [18], unlike BiFC and förster resonance energy transfer, it has limited spatial resolution at the subcellular level. Also, due to the use of protoplasts, our SFLC system can not be employed in those PPI studies where the cell wall or a tissue context is required. In addition, due to the high sensitivity and quantitative nature of the SFLC assay, co-expression of any two proteins fused with FLucN and FLucC, respectively, would result in a readout when measured by the luminometer. Since a clear cutoff value for positive PPIs is lacking for the assay, it is ambiguous whether a low but reproducible restored luciferase activity stands for a *bona fide* interaction or a relatively higher background. Therefore, the SFLC assay is more suitable for large-scale binary PPI screens, and we emphasize that any putative interaction fished out by the assay with modest restored luciferase activity needs to be confirmed by an alternative method such as co-IP.

Successful detection of PPI by the SFLC assay requires appropriate experimental design in terms of the negative control and the fusion orientation of luciferase fragments. A caveat is that co-expression of FLucN and FLucC constructs alone would produce low but significant restored luciferase activity (relative luciferase activity between 0.1 and 0.2, data not shown), resembling the spontaneous association of the fragments of a fluorescent protein in BiFC [42]. However, when fused with a pair of unrelated proteins, these luciferase fragments only generated a background level of signal (relative luciferase activity below 0.1, Figure 2A) probably because the fusion partners posed steric constraints on the luciferase fragments to impede their “kiss and run” motion. Thus, a proper negative control for the SFLC assay is not FLucN and FLucC alone but one fused to a protein being tested and the other fused to a co-localized but unrelated protein. Actually, only such a control can truly mimic a SFLC test as both luciferase fragments are under fusion circumstances. Regarding of the fusion orientation of luciferase fragments, several studies on luciferase complementation in animals and plants all concluded that the fusion orientation between luciferase fragments and the proteins being tested could dramatically affect the restored luciferase activity [11], [19], [43]. Other studies on protein complementation assays (e.g., BiFC) in plants have also reached the same conclusion [44]. In addition, a different fusion orientation of a luciferase fragment to a transmembrane protein could deposit the luciferase fragment into distinct subcellular compartments, which would prevent the luciferase complementation for certain combinations. As such, if the steric information of the potential interface between the two proteins being tested is predictable, the two luciferase fragments should be fused close to the anticipated interface to facilitate the reconstitution. Otherwise, the combinations of four different orientations, namely FLucN-protein A/FLucC-protein B, FLucN-protein A/protein B-FLucC, protein A-FLucN/FLucC-protein B, and protein A-FLucN/protein B-FLucC, should be comprehensively evaluated.

### Binary PPI screen among ARF and Aux/IAA proteins supports current auxin signaling model

Auxin signaling is central to plant growth and development [26]. It is thought that generic auxin signals are translated into specific gene expression through regulated PPIs between ARFs and Aux/IAAs, and between two ARFs. The former determines which ARF is to be inactivated [45], and the latter affects the promoter targeting of ARFs [46]. The Arabidopsis genome encodes 22 full-length ARFs and 29 Aux/IAAs [29], resulting in a huge complexity in specificity regulation of auxin responses within developmental and environmental contexts. So far, interaction studies between these proteins were mostly carried out in heterologous systems such as Y2H [26]-[28]. It is desirable to confirm these PPIs characterized in Y2H by *in vivo* PPI analysis in plant cells.

In this study, we comprehensively evaluated 96 binary PPIs between 8 ARFs and 12 Aux/IAAs by the SFLC assay in Arabidopsis mesophyll protoplasts. Among the 13 positive PPIs characterized previously in Y2H, 77% of them (10 PPIs) were found to have significant restored luciferase activity in plant cells using our SFLC assay (Table S3). However, two positive PPIs in Y2H, ARF1/IAA17 [31] and ARF5/IAA3 [45], could not be recovered in the SFLC assay. It is noteworthy that indirect evidence from gene repression assay *in planta* has suggested no interaction between ARF1 and IAA17 [24]. IAA3, albeit with high abundance in plant cells, was identified earlier as a rather weak transcription repressor [38], suggesting its marginal interaction with ARF transcription activators such as ARF5. Both *in planta* studies supported our SFLC data when the results from the SFLC assay and Y2H were in discrepancy. Compared to a very recently released Y2H data set covering the same 96 ARF and Aux/IAA interactions [28], over 60% of the ARF and Aux/IAA combinations showed consistent PPI results in both assays and most of the inconsistent results involved ARF5, ARF6, and ARF9 (data not shown). Again, in most of the cases where the SFLC assay and Y2H arrived at conflicting results, positive interactions were found in Y2H while negative results were obtained in the SFLC assay. Interestingly, in another recent work Shen *et al.* also noticed that many positive interactions between rice ARFs and Aux/IAAs identified by Y2H could not be reproduced in plants using the luciferase complementation imaging (LCI) assay [27]. It is highly likely that the interactions between ARFs and Aux/IAAs may be more specific and tightly regulated in plants than in yeast. Therefore, as an *in planta* system, the SFLC assay may be more accurate in pinpointing *in vivo* PPIs for plant proteins than Y2H.

We also systematically assessed 36 pairwise interactions between the 8 ARF proteins using the SFLC assay. Interestingly, compared to the ARF-ARF interactions identified earlier by Y2H (Table S3, [28]), more positive interactions were uncovered by the SFLC assay (Table S3 and data not shown). As both assays used the same CTD of ARFs in the tests, the reason behind these inconsistent observations was enigmatic. Future PPI analysis using a different *in planta* method (e.g., co-IP) will be necessary to solve this discrepancy.

Previous studies and ours suggested that the cellular repertoire of ARFs and Aux/IAAs consists of 6 types of dimers, namely ARF activator dimer, ARF repressor dimer, ARF activator-ARF repressor dimer, ARF activator-Aux/IAA dimer, ARF repressor-Aux/IAA dimer, and Aux/IAA dimer. Since different dimerizations occur through the same domain (i.e., CTD) in both ARF and Aux/IAA proteins [26], one protein would have to prefer a dimerization with higher PPI intensity. As reflected by the quantitative SFLC data, the ARF activator-Aux/IAA dimers (Figure 3A) and the ARF repressor dimers (Figure 4A) are dominant over other dimers due to their relatively stronger PPIs when the Aux/IAAs are in considerable abundances under low auxin conditions. The consequence of these PPIs would be the repression of auxin-responsive gene expression either through inhibition of ARF activators by Aux/IAA association or through binding of ARF repressors to the auxin-responsive elements in the promoters [26]. Under high auxin condition, degradation of Aux/IAA proteins may allow the released ARF activator monomer to form activator dimers [47]. It is not clear yet whether auxin-responsive gene activation requires the dimerization of ARF activators.

It should be stressed that the primary goal of the ARF-Aux/IAA interaction tests carried out in this study was to demonstrate the proof of concept of applying our SFLC system in large-scale PPI analysis and to establish a streamlined protocol. Thus, we made no efforts to fully characterize these interactions under elevated auxin conditions or using artificially stabilized Aux/IAA mutants. Also, we could not exclude the possibilities that other domains in the ARF protein may potentially interfere with the interaction between its CTD and Aux/IAAs [27], and that some of the binary PPIs tested here may be under artificial situations as the two proteins may not be co-expressed in the same cell type *in planta*. Unlike in Y2H, these particular limitations can be minimized in the SFLC assay by using the full-length ARFs in PPI tests, using the native promoter to drive gene expression, and using root or seedling protoplasts instead of mesophyll protoplasts for transient expression. Therefore, the SFLC assay reported here has the potential for depicting complete pictures of ARF-Aux/IAA interactions behind auxin signaling and of other PPI networks underlying complicated cellular processes in plant research.

## Materials and Methods

### Plant growth conditions

Wild-type Arabidopsis Col-0 plants were grown on either Metro-Mix 360 or Jiffy7 soil in a cycle of 12 hr light at 23°C followed by 12 hr dark at 20°C as described previously [48]. The light intensity of 75 µE m^-2^S^-1^ and the relative humidity of 65-75% were used for plant growth.

### Molecular cloning

All recombinant plasmids constructed in this study are listed in Table S4 and are available upon request. Standard molecular cloning protocols were followed for plasmid construction. Briefly, the coding sequence of the N-terminal fragment (FLucN, aa1-aa398) or the C-terminal fragment (FLucC, aa394-aa550) of firefly luciferase was PCR amplified, digested and inserted into the *Bam*HI/*Not*I sites of the pAN vector to obtain pcFLucN and pcFLucC plasmids (Figure S1), or inserted into the *Nhe*I/*Bam*HI sites of the pAN vector to produce pnFLucN and pnFLucC plasmids (Figure S1). These plasmids allow the fusion of any gene of interest with the FLucN or FLucC in either orientation as desired. In this study, the plasmids pcFLucN and pcFLucC were further opened with *Nhe*I/*Bam*HI digestion, and the genes being tested were inserted after digestion with either *Spe*I or *Nhe*I or *Xba*I at the 5′ end and with either *Bam*HI or *Bgl*II at the 3′ end.

### Protoplast isolation and transfection

Protoplasts were isolated from 4-5 weeks old plants according to the Sheen lab protocol ([48], http://genetics.mgh.harvard.edu/sheenlab). Briefly, 24 pieces of well-expanded green leaves (about 1.5-cm in length) were cut into 1-mm strips with a clean razor blade and were digested in 10 ml filtered enzyme solution (1.5% Cellulase R10, 0.4% macerozyme R10, 0.4 M mannitol, 20 mM KCl, 20 mM MES, pH 5.7, 10 mM CaCl_2_, 0.1% BSA) for 3 hr including the first 30 min for a vacuum infiltration step. After filtered through a piece of miracloth, protoplasts were pelleted by 2 min centrifugation at 1,500 rpm in a CL2 clinical centrifuge (Thermo Scientific) and were resuspended in 10 ml W5 solution (154 mM NaCl, 125 mM CaCl_2_, 5 mM KCl, 2 mM MES, pH 5.7). After resting on ice for at least 30 min, protoplasts were spun down by another 1 min centrifugation at 1,500 rpm in a CL2 centrifuge and were resuspended into 14 ml MMg solution (0.4 M mannitol, 15 mM MgCl_2_, 4 mM MES, pH 5.7) which roughly diluted the protoplasts into the optimal working concentration of 2×10^5^ cells per ml if no hemacytometer was available for precise cell counting. All plasmid DNA used for protoplast transfection were purified by CsCl gradient ultracentrifugation or homemade silica resin [49]. DNA transfection was carried out in a 2 ml round-bottom microcentrifuge tube where 100 µl protoplasts (2×10^4^ cells) were mixed well with 10 µg each of FLucN and FlucC constructs as well as 1 µg UBQ10::GUS plasmid, which was used as an internal control to normalize the transfection rate. A maximum of 20 transfection can be conducted at one time. 110 µl PEG solution (40% PEG, v/v, 0.2 M mannitol, 0.1 M CaCl_2_) was added to each tube and transfection was initiated sequencially by a gentle tapping at the tube bottom for 15 times. After a 5 min incubation at room temperature, transfection was terminated in the same order by adding 400 µl W5 solution and inverting the tube gently for 3 times. Transfected protoplasts were concentrated by centrifugation at 1,500 rpm for 2 min in a CL2 centrifuge and were resuspended in 30 µl W5 solution. The protoplasts were then transferred into 100 µl WI solution containing D-luciferin (0.5 M mannitol, 4 mM MES, pH 5.7, 20 mM KCl, 250 µg/ml D-luciferin) in a 96-well plate (black with white well). The samples were incubated on the lab bench for 6 hr before subsequent luminescence analysis.

### Luminescence analysis and GUS assay

The *in vivo* luminescence of each sample was recorded by a GloMax®-Multi microplate multimode reader (Promega) with the integration time set as 1 sec. It is worth mentioning that, to maximize the efficiency of HTS, one can use the Xenogen IVIS 100 system (Caliper Life Sciences) to quantitatively measure the luciferase activity. The latter is considered more sensitive than most of the commercial luminometers, and enables up to 3 plates to be assayed simultaneously and then repetitively at a high temporal resolution (30 sec interval), thereby enhancing the high-throughput and dynamic performance. However, a disadvantage of the IVIS system is that it is not currently coupled to robotic rails like typical luminometers for HTS. After the luminescence analysis, 100 µl of lysis solution (25 mM Tris-phosphate, pH 7.8, 2 mM DTT, 2 mM 1, 2-diaminocyclohexane-N,N,N',N'-tetraacetic acid, 10% glycerol, 1% Triton X-100) was immediately added to each well of the 96-well microplate using a 12-channel pipette. After covered with a Microseal B adhesive seal (Bio-Rad), the plate was shaken on a vortex mixer (VWR) at 675 rpm for 10 min. During this period, a fresh 96-well microplate was cooled down on ice and 50 µl of the MUG solution (0.5 mM MUG, 10 mM Tris-HCl, pH 8.0, 2 mM MgCl_2_) was added to each well using a 12-channel pipette. 5 µl of the protoplast lysate was then transferred from the lysate plate to the MUG containing plate. It is important to keep the MUG-containing plate at low temperature to prevent the start of GUS reaction for early mixed samples. The GUS reaction plate was then incubated at 37°C for 20 min and the reaction was terminated by quickly cooling down the plate in an ice-water bath. The GUS activity was subsequently measured on the same microplate reader.

### Western blot

The FLucN fusion proteins were blotted with mouse monoclonal antibody against the N-terminal 258 amino acids of firefly luciferase (NovusBio), while the FLucC fusion proteins were blotted with rabbit polyclonal antibody against the C-terminal 300 amino acids of firefly luciferase (Santa Cruz). After blotted with corresponding HRP-conjugated secondary antibody, the protein signal was visualized with the SuperSignal West Pico chemiluminescent kit (Thermo Scientific).

### Co-immunoprecipitation

Co-IP was performed as described previously [50]. Briefly, 100 µg of IAA7-FLAG or 20 µg of IAA19-FLAG plasmid was used to co-transfect 1 ml protoplasts (5 × 10^5^ cells) with 100 µg of indicated ARFCTD-FLucC plasmid. After 6 hr expression, the cell pellet was lysed in 0.5 ml IP lysis buffer (10 mM HEPES, pH7.5, 100 mM NaCl, 1 mM EDTA, 10% glycerol, 0.5% Triton X-100, EDTA-free protease inhibitor cocktail from Roche) by vigorous vortexing for 1 min. After centrifugation at maximal speed for 10 min at 4°C, the supernatant was incubated with 10 µl anti-FLAG M2 agarose beads (Sigma) for 3 hr. The beads were pelleted and washed 5 times with IP washing buffer (10 mM HEPES, pH7.5, 100 mM NaCl, 1 mM EDTA, 10% glycerol, 0.1% Triton X-100, EDTA-free protease inhibitor cocktail) and once with 50 mM Tris-HCl, pH7.5. The IP fraction was obtained by boiling the beads in 40 µl 1×SDS-PAGE loading buffer and its composition was dissected by western blot using anti-FLAG (Roche) and anti-FLucC antibodies.

### Z-factor determination

The Z-factor of the SFLC assay was calculated step by step as previously described [30]. Briefly, (i) compute the threshold value for negative controls as the mean signal of the negative controls plus three times their standard deviation; (ii) compute the threshold value for positive controls as the mean signal of the positive controls minus three times their standard deviation; (iii) compute the difference between the two thresholds as the “separation band (S)” of the assay; (iv) compute the difference between the two means as the “dynamic range (R)” of the assay; (v) compute the Z factor as S/R.

## Supporting Information

Figure S1 **Diagram of the split firefly luciferase expression vectors.** The plasmids pcFLucN and pcFLucC allow expression of the gene of interest with a C-terminal FLucN or FLucC fusion. The asterisk marks the stop codon in these vectors. The plasmids pnFLucN and pnFLucC allow expression of the gene of interest with an N-terminal FLucN or FLucC fusion. The start codon in both vectors has been indicated. The labeled restriction enzymes have single cut on the vector at the indicated site. *Bam*HI site is in frame with the FLucN or FLucC coding sequence in all vectors. All vectors contain a double *35S* promoter (*d35S*) and a *Nos* terminator (*Nos*) for transient expression, and an in-frame coding sequence for a GGSGGGGSGG linker (colored in orange) between the gene and the coding sequence of FLucN or FLucC.
(TIF)

Figure S2 **ARF5CTD could be localized in the nucleus.** A significant fraction of ARF5CTD-mCherry was detected in the nucleus labeled by the nuclear targeting GFP (NLS-GFP) after transient expression in Arabidopsis mesophyll protoplasts. The scale bar = 10 µm.
(TIF)

Table S1 **Quantitative analysis of interaction network between 12 Aux/IAA and 8 ARF proteins by split firefly luciferase complementation.**
(DOC)

Table S2 **Quantitative analysis of homo- and hetero-dimerizations between 8 ARFs by split firefly luciferase complementation.**
(DOC)

Table S3 **Comparison of the ARF-Aux/IAA and ARF-ARF interactions tested previously by Y2H and in this study by SFLC.**
(DOC)

Table S4 **Summary of the plasmids constructed in this study.**
(DOC)
