# Supplementary figures and images for: Large-Scale Protein-Protein Interaction Analysis in Arabidopsis Mesophyll Protoplasts by Split Firefly Luciferase Complementation
Source: PLoS One. 2011 Nov 9;6(11):e27364. doi: 10.1371/journal.pone.0027364 (PMC3212559; doi:10.1371/journal.pone.0027364)

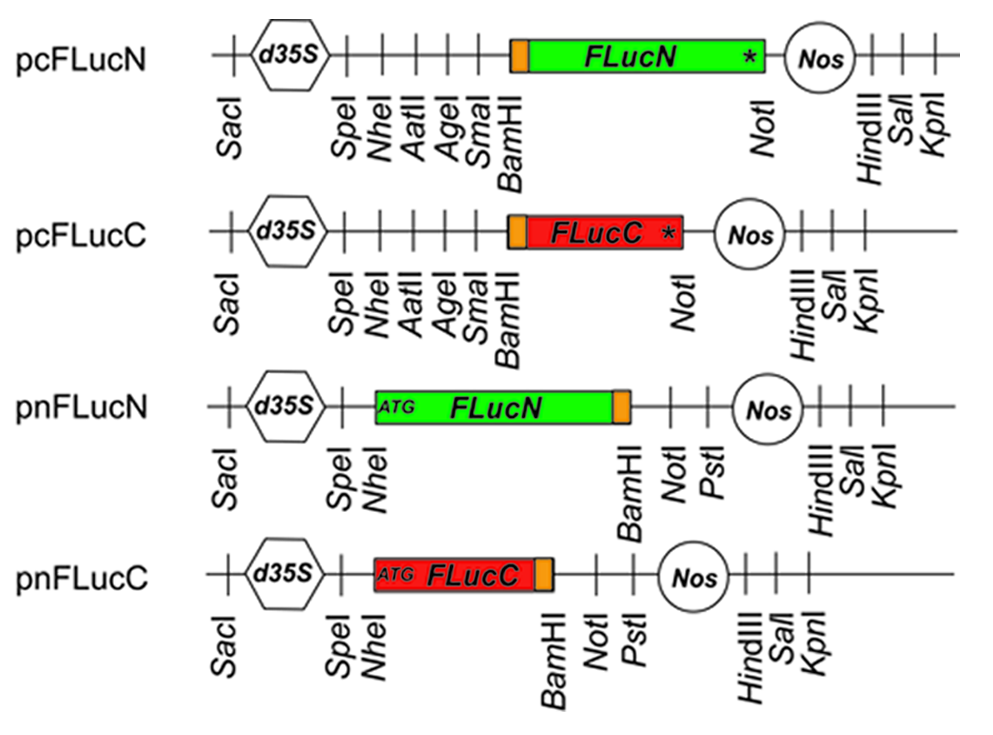

Supplement: Figure S1 — Diagram of the split firefly luciferase expression vectors. The plasmids pcFLucN and pcFLucC allow expression of the gene of interest with a C-terminal FLucN or FLucC fusion. The asterisk marks the stop codon in these vectors. The plasmids pnFLucN and pnFLucC allow expression of the gene of interest with an N-terminal FLucN or FLucC fusion. The start codon in both vectors has been indicated. The labeled restriction enzymes have single cut on the vector at the indicated site. BamHI site is in frame with the FLucN or FLucC coding sequence in all vectors. All vectors contain a double 35S promoter (d35S) and a Nos terminator (Nos) for transient expression, and an in-frame coding sequence for a GGSGGGGSGG linker (colored in orange) between the gene and the coding sequence of FLucN or FLucC. (TIF) [file pone.0027364.s001.tif]

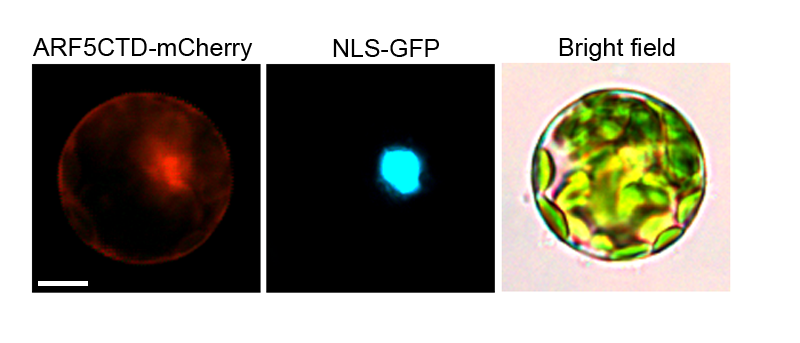

Supplement: Figure S2 — ARF5CTD could be localized in the nucleus. A significant fraction of ARF5CTD-mCherry was detected in the nucleus labeled by the nuclear targeting GFP (NLS-GFP) after transient expression in Arabidopsis mesophyll protoplasts. The scale bar = 10 µm. (TIF) [file pone.0027364.s002.tif]
