# Supplementary material for: Large-Scale Protein-Protein Interaction Analysis in Arabidopsis Mesophyll Protoplasts by Split Firefly Luciferase Complementation
Source: PLoS One. 2011 Nov 9;6(11):e27364. doi: 10.1371/journal.pone.0027364 (PMC3212559; doi:10.1371/journal.pone.0027364)
Supplement: Table S1 — Quantitative analysis of interaction network between 12 Aux/IAA and 8 ARF proteins by split firefly luciferase complementation. (DOC) [file pone.0027364.s003.doc]

| **FLucC**  **FLucN** | **ARF1***^a^* | **ARF4** | **ARF5** | **ARF6** | **ARF9** | **ARF10** | **ARF12** | **ARF18** |
| --- | --- | --- | --- | --- | --- | --- | --- | --- |
| **IAA1** | 0.014 *^b^*  ±0.002 | 0.038  ±0.010 | 0.589  ±0.059 | 0.035  ±0.004 | 0.017  ±0.004 | 0.010  ±0.001 | 0.015  ±0.001 | 0.022  ±0.005 |
| **IAA3** | 0.025  ±0.004 | 0.049  ±0.015 | 0.099  ±0.020 | 0.025  ±0.005 | 0.025  ±0.003 | 0.009  ±0.006 | 0.015  ±0.004 | 0.032  ±0.017 |
| **IAA6** | 0.064  ±0.006 | 0.292  ±0.060 | 0.802  ±0.139 | 0.116  ±0.024 | 0.144  ±0.022 | 0.031  ±0.011 | 0.041  ±0.005 | 0.214  ±0.042 |
| **IAA7** | 0.002  ±0.001 | 0.002  ±0.001 | 0.002  ±0.001 | 0.001  ±0.001 | 0.002  ±0.001 | 0.001  ±0.001 | 0.001  ±0.001 | 0.001  ±0.001 |
| **IAA9** | 0.044  ±0.004 | 0.179  ±0.031 | 0.560  ±0.037 | 0.092  ±0.031 | 0.081  ±0.026 | 0.025  ±0.009 | 0.024  ±0.004 | 0.139  ±0.024 |
| **IAA12** | 0.175  ±0.007 | 0.095  ±0.025 | 0.411  ±0.035 | 0.055  ±0.011 | 0.089  ±0.013 | 0.024  ±0.006 | 0.079  ±0.005 | 0.127  ±0.008 |
| **IAA13** | 0.140  ±0.025 | 0.089  ±0.009 | 0.432  ±0.037 | 0.068  ±0.001 | 0.087  ±0.018 | 0.025  ±0.004 | 0.082  ±0.004 | 0.105  ±0.012 |
| **IAA14** | 0.012  ±0.001 | 0.077  ±0.022 | 0.250  ±0.042 | 0.035  ±0.005 | 0.028  ±0.004 | 0.007  ±0.001 | 0.009  ±0.001 | 0.061  ±0.010 |
| **IAA17** | 0.021  ±0.003 | 0.080  ±0.018 | 0.248  ±0.043 | 0.036  ±0.006 | 0.037  ±0.008 | 0.011  ±0.002 | 0.023  ±0.005 | 0.051  ±0.007 |
| **IAA18** | 0.247  ±0.063 | 0.124  ±0.046 | 0.360  ±0.045 | 0.086  ±0.004 | 0.086  ±0.022 | 0.032  ±0.007 | 0.063  ±0.044 | 0.229  ±0.046 |
| **IAA19** | 0.093  ±0.026 | 0.412  ±0.132 | 0.594  ±0.031 | 0.097  ±0.025 | 0.154  ±0.050 | 0.047  ±0.022 | 0.048  ±0.011 | 0.174  ±0.046 |
| **IAA28** | 0.735  ±0.034 | 1.026  ±0.200 | 1.000  ±0.000 | 0.305  ±0.025 | 0.431  ±0.125 | 0.229  ±0.039 | 0.192  ±0.060 | 0.588  ±0.100 |
| *^a^*Only the C-terminal domain (CTD) of ARFs was used in the SFLC assay.  *^b^*The value corresponds to a relative restored firefly luciferase activity, which was generated by standardizing against that of ARF5CTD-FLucC and IAA28-FLucN interaction. At least three biological replicates were performed for each Aux/IAA-ARF combination. | | | | | | | | |

**Table S1** Quantitative analysis of interaction network between 12 Aux/IAA and 8 ARF proteins by split firefly luciferase complementation
