# Supplementary material for: Large-Scale Protein-Protein Interaction Analysis in Arabidopsis Mesophyll Protoplasts by Split Firefly Luciferase Complementation
Source: PLoS One. 2011 Nov 9;6(11):e27364. doi: 10.1371/journal.pone.0027364 (PMC3212559; doi:10.1371/journal.pone.0027364)
Supplement: Table S2 — Quantitative analysis of homo- and hetero-dimerizations between 8 ARFs by split firefly luciferase complementation. (DOC) [file pone.0027364.s004.doc]

| **FLucC**  **FLucN** | **ARF1***^a^* | **ARF4** | **ARF5** | **ARF6** | **ARF9** | **ARF10** | **ARF12** | **ARF18** |
| --- | --- | --- | --- | --- | --- | --- | --- | --- |
| **ARF1** | 0.649 *^b^*  ±0.047 |  |  |  |  |  |  | 0.840  ±0.245 |
| **ARF4** | 0.379  ±0.076 | 0.377  ±0.138 |  |  |  |  |  | 0.303  ±0.077 |
| **ARF5** | 0.203  ±0.100 | 0.186  ±0.033 | 0.211  ±0.074 |  |  |  |  | 0.288  ±0.114 |
| **ARF6** | 0.119  ±0.045 | 0.157  ±0.031 | 0.176  ±0.043 | 0.089  ±0.039 |  |  |  | 0.117  ±0.001 |
| **ARF9** | 0.819  ±0.222 | 0.318  ±0.040 | 0.389  ±0.060 | 0.068  ±0.039 | 0.397  ±0.076 |  |  | 0.631  ±0.124 |
| **ARF10** | 0.250  ±0.042 | 0.366  ±0.072 | 0.287  ±0.028 | 0.188  ±0.072 | 0.178  ±0.042 | 0.235  ±0.128 |  | 0.205  ±0.064 |
| **ARF12** | 0.407  ±0.040 | 0.493  ±0.016 | 0.431  ±0.089 | 0.140  ±0.013 | 0.445  ±0.068 | 0.120  ±0.021 | 0.209  ±0.022 | 0.255  ±0.050 |
| **ARF18** | 0.731  ±0.009 | 0.343  ±0.033 | 0.450  ±0.046 | 0.086  ±0.039 | 0.523  ±0.224 | 0.123  ±0.043 | 0.263  ±0.039 | 0.501  ±0.128 |
| *^a^* Only the C-terminal domain (CTD) of ARFs was used in the SFLC assay.  *^b^*The value corresponds to a relative restored firefly luciferase activity, which was generated by standardizing against that of ARF5CTD-FLucC and IAA28-FLucN interaction. At least three biological replicates were performed for each ARF-ARF combination. | | | | | | | | |

**Table S2** Quantitative analysis of homo- and hetero-dimerization between 8 ARFs by split firefly luciferase complementation
