# Supplementary material for: Large-Scale Protein-Protein Interaction Analysis in Arabidopsis Mesophyll Protoplasts by Split Firefly Luciferase Complementation
Source: PLoS One. 2011 Nov 9;6(11):e27364. doi: 10.1371/journal.pone.0027364 (PMC3212559; doi:10.1371/journal.pone.0027364)
Supplement: Table S3 — Comparison of the ARF-Aux/IAA and ARF-ARF interactions tested previously by Y2H and in this study by SFLC. (DOC) [file pone.0027364.s005.doc]

**Table S3** Comparison of the ARF-Aux/IAA and ARF-ARF interactions tested previously by Y2H and in this study by SFLC

| **Combination** | **Y2H result/Reference** | **Result in this study***^a^* |
| --- | --- | --- |
| ARF1/IAA12 | Positive/[1] | **++** |
| ARF1/IAA13 | Positive/[1] | **++** |
| ARF1/IAA17 | Positive/[2] | **+***^b,c^* |
| ARF5/IAA1 | Positive/[3] | **++++++** |
| ARF5/IAA3 | Positive/[4] | **+***^d^* |
| ARF5/IAA6 | Positive/[3] | **++++++++** |
| ARF5/IAA12 | Positive/[4]; [5] | **+++++** |
| ARF5/IAA13 | Positive/[5] | **+++++** |
| ARF5/IAA14 | Positive/[6] | **+++** |
| ARF5/IAA17 | Positive/[2] | **+++** |
| ARF5/IAA19 | Positive/[3] | **++++++** |
| ARF5/IAA28 | Positive/[7] | **++++++++** |
| ARF6/IAA1 | Positive/[8] | **+** |
| ARF1/ARF1 | Positive/[2] | **+++++++** |
| ARF5/ARF1 | Positive/[2] | **+++** |
| ARF5/ARF4 | Negative/[9] | **++** |
| ARF5/ARF5 | Positive/[3]; [9] | **+++** |
| ARF5/ARF6 | Negative/[9] | **++** |
| ARF5/ARF9 | Negative/[9] | **++++** |
| *^a^*68% (13/19) of the reported Y2H results in the literature are consistent with the SFLC results in this study  *^b^*Interaction results in this study that are inconsistent with the previous Y2H results are highlighted in red  *^c^*Gene repression assay *in planta* suggested no interaction between ARF1 and IAA17 [10], supporting the SFLC result in this study  *^d^*Gene repression assay *in planta* suggested no or very weak interaction between ARF5 and IAA3 [11], supporting the SFLC result in this study | | |

References

1. Ulmasov T, Murfett J, Hagen G, Guilfoyle TJ (1997) Aux/IAA proteins repress expression of reporter genes containing natural and highly active synthetic auxin response elements. Plant Cell 9: 1963-1971.
2. Ouellet F, Overvoorde PJ, Theologis A (2001) IAA17/AXR3: biochemical insight into an auxin mutant phenotype. Plant Cell 13: 829-841.
3. Tatematsu K, Kumagai S, Muto H, Sato A, Watahiki MK, et al. (2004) *MASSUGU2* encodes Aux/IAA19, an auxin-regulated protein that functions together with the transcriptional activator NPH4/ARF7 to regulate differential growth responses of hypocotyl and formation of lateral roots in *Arabidopsis thaliana*. Plant Cell 16: 379-393.
4. Weijers D, Benkova E, Jäger KE, Schlereth A, Hamann T, et al. (2005) Developmental specificity of auxin response by pairs of ARF and Aux/IAA transcriptional regulators. EMBO J 24: 1874-1885.
5. Hamann T, Benkova E, Baurle I, Kientz M, Jürgens G (2002) The Arabidopsis *BODENLOS* gene encodes an auxin response protein inhibiting *MONOPTEROS*-mediated embryo patterning. Genes Dev 16: 1610-1615.
6. Fukaki H, Nakao Y, Okushima Y, Theologis A, Tasaka M (2005) Tissue-specific expression of stabilized SOLITARY-ROOT/IAA14 alters lateral root development in Arabidopsis. Plant J 44: 382-395.
7. De Rybel B, Vassileva V, Parizot B, Demeulenaere M, Grunewald W, et al. (2010) A novel aux/IAA28 signaling cascade activates GATA23-dependent specification of lateral root founder cell identity. Curr Biol 20: 1697-1706.
8. Kim J, Harter K, Theologis A (1997) Protein-protein interactions among the Aux/IAA proteins. Proc Natl Acad Sci USA 94: 11786-11791.
9. Hardtke CS, Ckurshumova W, Vidaurre DP, Singh SA, Stamatiou G, et al. (2004) Overlapping and non-redundant functions of the Arabidopsis auxin response factors MONOPTEROS and NONPHOTOTROPIC HYPOCOTYL 4. Development 131: 1089-1100.
10. Tiwari SB, Hagen G, Guilfoyle TJ (2003) The roles of auxin response factor domains in auxin-responsive transcription. Plant Cell 15: 533-543.
11. Maraschin FdosS, Memelink J, Offringa R (2009) Auxin-induced, SCF(TIR1)-mediated poly-ubiquitination marks AUX/IAA proteins for degradation. Plant J 59: 100-109.
