# Supplementary material for: Large-Scale Protein-Protein Interaction Analysis in Arabidopsis Mesophyll Protoplasts by Split Firefly Luciferase Complementation
Source: PLoS One. 2011 Nov 9;6(11):e27364. doi: 10.1371/journal.pone.0027364 (PMC3212559; doi:10.1371/journal.pone.0027364)
Supplement: Table S4 — Summary of the plasmids constructed in this study. (DOC) [file pone.0027364.s006.doc]

**Table S4** Summary of the plasmids constructed in this study

| **Plasmid name** | **Gene of interest** | **Luciferase fragment** | **Protein product***^a^* |
| --- | --- | --- | --- |
| pcFLucN | N/A*^b^* | FLuc*^c^* aa1-398 | N/A |
| pcFLucC | N/A | FLuc aa394-550 | N/A |
| pnFLucN | N/A | FLuc aa1-398 | N/A |
| pnFLucC | N/A | FLuc aa394-550 | N/A |
| pnFLucN-AtFRB | AtFRB | FLuc aa1-398 | FLucN-AtFRB |
| pnFLucC-HsFKBP | HsFKBP | FLuc aa394-550 | FLucC-HsFKBP |
| pcMYB30-FLucN | MYB30 | FLuc aa1-398 | MYB30-FLucN |
| pcIAA1-FLucN | IAA1 | FLuc aa1-398 | IAA1-FLucN |
| pcIAA3-FLucN | IAA3 | FLuc aa1-398 | IAA3-FLucN |
| pcIAA6-FLucN | IAA6 | FLuc aa1-398 | IAA6-FLucN |
| pcIAA7-FLucN | IAA7 | FLuc aa1-398 | IAA7-FLucN |
| pcIAA9-FLucN | IAA9 | FLuc aa1-398 | IAA9-FLucN |
| pcIAA12-FLucN | IAA12 | FLuc aa1-398 | IAA12-FLucN |
| pcIAA13-FLucN | IAA13 | FLuc aa1-398 | IAA13-FLucN |
| pcIAA14-FLucN | IAA14 | FLuc aa1-398 | IAA14-FLucN |
| pcIAA17-FLucN | IAA17 | FLuc aa1-398 | IAA17-FLucN |
| pcIAA18-FLucN | IAA18 | FLuc aa1-398 | IAA18-FLucN |
| pcIAA19-FLucN | IAA19 | FLuc aa1-398 | IAA19-FLucN |
| pcIAA28-FLucN | IAA28 | FLuc aa1-398 | IAA28-FLucN |
| pcARF1-FLucN | ARF1 aa537-665 | FLuc aa1-398 | ARF1CTD-FLucN |
| pcARF4-FLucN | ARF4 aa660-788 | FLuc aa1-398 | ARF4CTD-FLucN |
| pcARF5-FLucN | ARF5 aa788-902 | FLuc aa1-398 | ARF5CTD-FLucN |
| pcARF6-FLucN | ARF6 aa791-935 | FLuc aa1-398 | ARF6CTD-FLucN |
| pcARF9-FLucN | ARF9 aa518-638 | FLuc aa1-398 | ARF9CTD-FLucN |
| pcARF10-FLucN | ARF10 aa575-693 | FLuc aa1-398 | ARF10CTD-FLucN |
| pcARF12-FLucN | ARF12 aa506-593 | FLuc aa1-398 | ARF12CTD-FLucN |
| pcARF18-FLucN | ARF18 aa484-602 | FLuc aa1-398 | ARF18CTD-FLucN |
| pcIAA1-FLucC | IAA1 | FLuc aa394-550 | IAA1-FLucC |
| pcIAA3-FLucC | IAA3 | FLuc aa394-550 | IAA3-FLucC |
| pcIAA6-FLucC | IAA6 | FLuc aa394-550 | IAA6-FLucC |
| pcIAA7-FLucC | IAA7 | FLuc aa394-550 | IAA7-FLucC |
| pcIAA9-FLucC | IAA9 | FLuc aa394-550 | IAA9-FLucC |
| pcIAA12-FLucC | IAA12 | FLuc aa394-550 | IAA12-FLucC |
| pcIAA13-FLucC | IAA13 | FLuc aa394-550 | IAA13-FLucC |
| pcIAA14-FLucC | IAA14 | FLuc aa394-550 | IAA14-FLucC |
| pcIAA17-FLucC | IAA17 | FLuc aa394-550 | IAA17-FLucC |
| pcIAA18-FLucC | IAA18 | FLuc aa394-550 | IAA18-FLucC |
| pcIAA19-FLucC | IAA19 | FLuc aa394-550 | IAA19-FLucC |
| pcIAA28-FLucC | IAA28 | FLuc aa394-550 | IAA28-FLucC |
| pcARF1-FLucC | ARF1 aa537-665 | FLuc aa394-550 | ARF1CTD-FLucC |
| pcARF4-FLucC | ARF4 aa660-788 | FLuc aa394-550 | ARF4CTD-FLucC |
| pcARF5-FLucC | ARF5 aa788-902 | FLuc aa394-550 | ARF5CTD-FLucC |
| pcARF6-FLucC | ARF6 aa791-935 | FLuc aa394-550 | ARF6CTD-FLucC |
| pcARF9-FLucC | ARF9 aa518-638 | FLuc aa394-550 | ARF9CTD-FLucC |
| pcARF10-FLucC | ARF10 aa575-693 | FLuc aa394-550 | ARF10CTD-FLucC |
| pcARF12-FLucC | ARF12 aa506-593 | FLuc aa394-550 | ARF12CTD-FLucC |
| pcARF18-FLucC | ARF18 aa484-602 | FLuc aa394-550 | ARF18CTD-FLucC |
| *^a^*A double GGSGG peptide linker was generated between the gene product and the luciferase fragment  *^b^*N/A, not applicable  *^c^*FLuc, firefly luciferase | | | |
